# CD147 promotes collective invasion through cathepsin B in hepatocellular carcinoma

**DOI:** 10.1186/s13046-020-01647-2

**Published:** 2020-07-29

**Authors:** Shi-Jie Wang, Dong Chao, Wei Wei, Gang Nan, Jia-Yue Li, Fen-Ling Liu, Ling Li, Jian-Li Jiang, Hong-Yong Cui, Zhi-Nan Chen

**Affiliations:** 1 National Translational Science Center for Molecular Medicine, Department of Cell Biology, Fourth Military Medical University, Xi’an, 710032 P. R. China; 2 Department of thoracic surgery, the 940th hospital of joint logistics support force of Chinese People’s Liberation Army, Lanzhou, 730050 P. R. China

**Keywords:** Hepatocellular carcinoma, Collective invasion, CD147, Cathepsin B

## Abstract

**Background:**

Mounting evidence suggests that solid tumors display the features of collective invasion, however, the molecular mechanisms are far from clear. This study aims to verify the role and the underlying mechanisms of CD147 in collective invasion in hepatocellular carcinoma.

**Methods:**

Immunostaining was used to analyze human hepatocellular carcinoma specimens and three-dimensional cultures. Three-dimensional invasion model was established to mimic in vivo invasion. RNA-sequencing was used to identify downstream effectors.

**Results:**

Human hepatocellular carcinoma underwent collective invasion and CD147 was observed to be upregulated at the invasive front of tumor cell groups. CD147 was demonstrated to promote collective invasion using the modified three-dimensional invasion model, which recapitulated the main features of collective invasion. Through transcriptome analysis and enzyme activity assay, we found that CD147 enhanced cathepsin B expression and activity. Upregulated cathepsin B in hepatocellular carcinoma cells facilitated migration and invasion, which mediated CD147-induced invasive phenotype in hepatocellular carcinoma. In terms of mechanism, we found that CD147 promoted cathepsin B transcription by activating β-catenin signaling as a result of reduced GSK-3β expression. Furthermore, we found that elevated expression of CD147 as well as cathepsin B were correlated with poor prognosis in patients with hepatocellular carcinoma.

**Conclusions:**

CD147 promotes hepatocellular carcinoma cells collective invasion via upregulating cathepsin B expression and targeting CD147 would be valuable for the development of novel therapeutic modalities against invasion and metastasis of cancer.

## Background

Liver cancer ranks the fourth leading cause of cancer-related death worldwide [1]. Hepatocellular carcinoma (HCC) accounts for about 85–90% of all primary liver malignancies and is refractory to nearly all currently available anti-cancer therapies. HCC is typically diagnosed late in the disease progression when treatment options are limited, causing disease-free survival rates to remain dismal [2]. Recent genetic profiling has shown that HCC displays a variety of mutations affecting p53 and the PI3K-AKT signaling [3]. However, targeting these for therapeutic interventions has remained difficult and largely unsuccessful. Thus, identification of additional factors or alternative methods for intervention remains a high priority.

Tumor metastasis is a complex multi-step process that is the sum of a series of cascade events in which tumor cells need to invade through the ECM, vascular endothelium and other structures. The interaction between tumor cells and microenvironment largely determines whether the tumor cells can metastasize. For example, the interaction between tumor cells and ECM is a key factor in determining whether tumor cells can reach adjacent blood vessels after separating from the primary site.

ECM is a complex macromolecular structure present in all organ and tissue types. It is rich in collagen and fibronectin, which contributes to maintain cell and tissue morphology, as an important place for intercellular interaction. In addition to providing basic physical support, ECM also establishes a biochemical and biomechanical environment that is critical to tissue homeostasis and differentiation. ECM is regarded as a constantly remodeling dynamic structure in which proteases are key molecules involved in ECM remodeling [4]. During tumor invasion and metastasis, the degradation of the ECM component is achieved primarily by the release of proteases from the tumor cells and stromal cells into the tumor microenvironment. The activated proteases degrade the matrix, thereby forming a localized solution that constitutes a tumor cell migration pathway. Under physiological conditions, these proteases are tightly regulated. In contrast, in malignant tumors, these regulatory mechanisms are missing or unbalanced. And studies have shown that there is a close relationship between the increased expression and activity of proteases and tumor invasion [5]. MMPs and cathepsins play a major role in the degradation of ECM protein components [6–8].

Another key factor affecting tumor cell invasion and metastasis is the change of cell motility triggered by tumor cell skeleton rearrangement. The ECM structure, including pore diameter, hardness, and other factors, regulates the way in which tumor cells move and invade. It is found that when the ECM pore size is smaller than the diameter of the nucleus, the tumor cells invade by a protease-dependent manner, and the ECM is degraded by the proteases. Here cells show a mesenchymal movement. When the ECM pore size is larger than the nucleus diameter, the tumor cells invade ECM via a non-protease-dependent manner, the cells show amoeba-like movement [9]. Mesenchymal cell movement and amoeboid-like cell movement can be converted to each other which mediated by Rac/WAVE2 signaling pathway and Rho /ROCK/p-mlc2 signaling pathway respectively [10]. Therefore, tumor cells as well as stromal cells can hydrolyze ECM by secretory proteases and promote ECM remodeling and tumor invasion, and remodeled ECM can also regulate the interconversion between mesenchymal and amoeboid movement and the process of tumor invasion.

Cancer cell invasion is commonly conceptualized as a single-cell process in loosely organized tissues like sarcomas, or in highly cohesive carcinomas that undergo an epithelial-to-mesenchymal transition (EMT), which is characterized by loss of epithelial adhesion and gain of mesenchymal features and activation of specific transcription factors, Twist, Zeb, Snail and Slug contributing to deficiency of E-cadherin expression. EMT is an important mechanism to empower epithelial cells into the motility that occurs during embryonic development and recurs in cancer and fibrosis. The role of EMT in metastasis is a longstanding source of debate. Mice models demonstrated that EMT program is dispensable for metastasis but contributes to chemoresistance in breast and pancreatic cancer [11, 12]. Mounting evidence suggests that the majority of solid tumors display features of collective invasion in which cells invade cohesively as a multicellular unit [13, 14]. The collective invasion of cancer cells has been verified over the past decade by intravital microscopy in a variety of experimental systems [15–17] and the systematic prospective analysis of primary tumor explants [18]. However, the molecular mechanisms underlying this process have been poorly understood.

CD147 is a single transmembrane glycoprotein that belongs to the immunoglobulin superfamily. CD147 was originally found to be able to induce fibroblast secretion of matrix metalloproteinases (MMPs) in lung cancer. Subsequent studies have shown that CD147 can induce a variety of cells, including fibroblasts, endothelial cells, macrophages, tumor cells and other cells to express MMPs (MMP-1, MMP-2, MMP-3, MMP-9, MT1-MMP and MT2, etc.), leading to extracellular matrix (ECM) remodeling, and thus promoting tumor cell invasion and metastasis [19, 20]. ECM remodeling and the change of cell motility are key factors affecting the invasion of tumor cells. In addition to inducing MMPs secretion, CD147 can also regulate cytoskeleton rearrangement and promote cell motility [21, 22]. Although numerous studies have confirmed that CD147-induced MMPs secretion plays an important role in the invasion and metastasis of HCC cells, our work in this study found that despite inhibition of MMPs activity, CD147 can still promote HCC cells invasion in a 3D model, suggesting that CD147 may regulate ECM remodeling and invasion of HCC cells via others effectors. Moreover, previous studies were based on 2D culture conditions and the effect and the underlying mechanisms of CD147-regulated ECM remodeling on tumor cell cytoskeleton rearrangement and cell motility in 3D culture model are far from clear.

In this study, we established a 3D invasion model and found that CD147 promoted 3D invasion of HCC cells regardless of MMPs activity. Protease expression profiling showed that CD147 may regulate a panel of proteases besides MMPs. Among these, expression of cathepsin B was significantly down-regulated in CD147 knockout/knockdown HCC cell lines, and there was a positive correlation between the expression of CD147 and cathepsin B in HCC tissues. Detailed analysis showed that CD147 promoted the HCC invasion and metastasis by upregulating the expression and activation of cathepsin B, facilitating extracellular hydrolysis and lysosomal degradation of collagen in ECM. We also found that CD147 increased cathepsin B transcription by activating β-catenin signaling via reduced GSK-3β. Furthermore, elevated expression of CD147 as well as cathepsin B correlated with poor prognosis in patients with HCC.

## Methods

### Reagents

TRIzol (15596026) was purchased from Sigma (St Louis, Missouri, USA). MMP inhibitor TAPI-2 (HY-100211) was obtained from MedChemExpress (Shanghai, China). Cathepsin B inhibitor CA-074 methyl ester (CA-074, S7420), GSK-3β inhibitor TWS119 (S1590), EGFR inhibitor PD153035 HCl (S1079), TGFR-1 inhibitor SB431542 (S1067), NF-κB inhibitor PDTC (S3633), STAT3 inhibitor WP1066 (S2796) and FAK inhibitor PF573228 (S2013) were purchased from Selleck (Shanghai, China). α-tubulin antibody (ab80779) was purchased from Abcam (Cambridge, MA, USA); Antibodies specific for E-cadherin (20874–1-AP), GSK-3β (22104–1-AP), lamin B (66095–1-Ig) were obtained from Proteintech (WuHan, China); Cathepsin B antibody (sc-6493) was obtained from Santa Cruz Biotechnology (CA, USA); β-catenin antibody (610153) was obtained from BD Biosciences (San Jose, CA, USA); goat anti-mouse IgG antibody (31430), goat anti-rabbit IgG antibody (31460), were purchased from Thermo Fisher Scientifc (Waltham, MA, USA); CD147-specific antibody was produced by our lab [23].

### Cell lines

Human hepatocellular carcinoma cell line Huh-7 was obtained from the Japanese Collection of Research Bioresources (JCRB, Osaka, Japan). HepG2 cells were obtained from Chinese Academy of Medical Sciences (Shanghai, China). HFF-1 cells were obtained from American Type Culture Collection (ATCC, USA). A Huh-7 CD147-KO (Huh-7 CD147^−/−^) cell line was generated using a CRISPR/Cas9 system as previously reported [24]. All cells were cultured at 37 °C, 5% CO_2_, in RPMI-1640 medium supplemented with 10% fetal bovine serum (FBS). All cells have been authenticated using short tandem repeat profiling.

### 3D invasion model

3D cultures referred to the method described by M. Horie et al. [25]. Briefly, collagen gels were prepared by mixing 0.5 ml cell suspension of HFF-1 (2.5 × 10^5^ cells) in FBS, 2.3 ml of type I collagen (Cell matrix type IA; 354,236, Corning), 670 μl of 5 × Dulbecco’s Modified Eagle’s medium (DMEM), and 330 μl of reconstitution buffer. The mixture (3 ml) was cast into each well of a 6-well culture plate. The solution was then allowed to polymerize at 37 °C for 30 min. For co-culture, cancer cells (2 × 10^5^ cells) were seeded on the surface of each gel and cultured in DMEM with 10% FBS. After incubation overnight, each gel was detached and cultured with growth medium for five additional days. Then exposed the gels to air by placing it on a filter in new plates with growth medium. After 5 days of the air–liquid interface culture, the gel was fixed in formalin solution and embedded in paraffin, and vertical sections (4 μm) were stained with hematoxylin and eosin.

### Cathepsin B activity assay

Cathepsin B activity was measured using the cathepsin B activity assay kit (KA0766, Abnova, Limerick, PA). 5 × 10^6^ cells were collected by centrifugation and lysed in 50 μl of pre-chilled CB Cell Lysis Buffer on ice for 10 min. Then centrifuge at top speed for 5 min, transfer the supernatant to a new tube. Add 50 μl of cell lysate to a 96-well plate. Add 50 μl of CB Reaction Buffer to each sample. Add 2 μl of the 10 mM CB Substrate Ac-RR-AFC (200 μM final concentration). Incubate at 37 °C for 1–2 h. Read samples in a fluorometer equipped with a 400 nm excitation filter and 505 nm emission filter (Infinite M200 Pro, TECAN, Austria).

### RNA sequencing

RNA sequencing was performed as previously described [23]. Briefly, total RNA was assessed for quantity and quality. Sequence libraries were generated and sequenced by CapitalBio Technology (Beijing, China). A NEB Next Ultra RNA Library Prep Kit for Illumina (NEB, Ipswich, MA, USA) was used to construct the libraries for sequencing. A NEB Next Poly(A) mRNA Magnetic Isolation Module (NEB, Ipswich, MA, USA) kit was used to enrich poly(A) tailed mRNA molecules from 1 μg total RNA. The mRNA was fragmented into ∼200 base pair pieces. First-strand cDNA was synthesized from the mRNA fragments using reverse transcriptase and random hexamer primers, after which second-strand cDNA was synthesized using DNA polymerase I and RNase H. The end of the cDNA fragments were subjected to an end repair process including the addition of a single “A” base, followed by ligation of the adapters. The resulting products were purified and enriched by PCR to amplify the library DNA. The final libraries were quantified using a KAPA Library Quantification kit (KAPA Biosystems, South Africa) and an Agilent 2100 Bioanalyzer. After qRT-PCR validation, the libraries were subjected to paired-end sequencing with a pair-end 150-base pair reading length on an Illumina HiSeq sequencer (Illumina). The clean reads were subsequently aligned to the reference genome and the processed reads from each sample were aligned using HISAT (Johns Hopkins University, USA). Cuffdiff was used to analyze the differentially expressed genes (DEGs) between samples. The standardization method of Cuffdiff is geometric, with per-condition and pooled as discrete model. Thousands independent statistical hypothesis tests were conducted on DEGs, separately, after which a *p* value was obtained that was corrected using an FDR method.

### Tissue specimens and immunohistochemistry

HCC tissue specimens were collected from the Department of Pathology (Eastern Hepatobiliary Surgery Hospital, which is affiliated with the Second Military Medical University) from 2008 to 2012 and were histologically confirmed by staining with hematoxylin and eosin (HE). All patients provided written informed consent, and the study was approved by the Hospital Ethics Committee.

Immunohistochemical (IHC) staining was performed on 5 μm tissue sections. Paraffin sections were dewaxed, followed by antigen retrieval with 10 μM citrate buffer at pH 6.0. The deparaffinized sections were treated with methanol containing 3% hydrogen peroxide for 15 min. After washing with PBS, the sections were incubated with blocking serum for 30 min. Then, the sections were incubated with primary antibody at 4 °C overnight. Following incubation, immunoperoxidase staining was conducted using a streptavidin-peroxidase kit (Zhongshan Jinqiao Co., Beijing, China) and the sections were treated with 3,3′-diaminobenzidine (Zhongshan Jinqiao Co., Beijing, China) to detect the target proteins. Hematoxylin was used to counterstain the nuclei. The expression level of the targets were independently evaluated by two senior pathologists according to the proportion and intensity of positive cells. The following criteria were used to score each specimen: 0 (no staining), 1 (any percentage with weak intensity or < 30% with intermediate intensity), 2 (> 30% with intermediate intensity or < 50% with strong intensity) or 3 (> 50% with strong intensity).

### Immunofluorescence assays

Immunofluorescence was performed as described previously [26]. Briefly, cells were harvested and allowed to attach for 24 h to cell culture dishes with glass bottoms (NEST Biotechnology Co., LTD.). After washing twice with PBS, the cells were fixed in paraformaldehyde in PBS, permeabilized with 0.1% Triton X-100, and blocked with 1% BSA in PBS for 1 h. The cells were first incubated with the indicated antibodies at 4 °C overnight, washed twice with PBS, and then incubated with the corresponding fluorescein-conjugated secondary antibodies for 1 h in the dark. Cell nuclei were stained with DAPI (Vector Labs). After washing, the cells were visualized using an A1R-A1 confocal laser microscope system (Nikon, Japan).

### Transfection and generation of stable cell lines

One day prior to transfection, 4 × 10^5^ cells were seeded per well in a 12-well plate in complete medium. Subsequent transfection was carried out using Lipofectamine 2000 (Invitrogen, Carlsbad, CA, USA) according to the manufacturer’s instructions. After transfection, the cells were subjected to selection in 10 μg/ml blasticidin (for shCTSB) or in 8 μg/ml puromycin (for shCD147 and CD147OE) for 2 weeks. Antibiotic resistant colonies were subsequently picked, pooled, and expanded for further analysis under selective conditions.

### Western blotting

Western blotting was performed as described previously [26]. Briefly, equal amounts of protein were separated by denaturing SDS-PAGE and transfered to polyvinylidene fluoride (PVDF) microporous membranes (Millipore, Boston, MA). Next, the resulting blots were blocked with 5% nonfat milk in TBS/0.5‰ Tween (TBS-T). The primary antibodies were diluted in TBS-T, and the blots were incubated with these antibodies at 4 °C overnight followed by washing in TBS-T and incubation with HRP-conjugated secondary antibodies for 1 h at room temperature. Signal detection was conducted using a ChemiDoc™ Touch Imaging System and analyzed using Image Lab™ Software (Bio-Rad, CA, USA).

### Scratch wound healing assay

In vitro scratch wound-healing assays were performed as described previously [26]. Briefly, 24 h after treatment, the cells were harvested, seeded in 24-well plates and grown until confluence. Next, a pipette was used to scratch (‘wound’) the monolayer after which the remaining cells were washed with serum-free medium. Subsequently, photomicrographs were taken at various time points.

### In vivo metastasis assay

Immunodeficient nude mice (strain BALB/c, 6–8 weeks) were obtained from the Institute of Laboratory Animal Sciences, Chinese Academy of Medical Sciences, and the animal study was approved by the Animal Care and Use Committee of Fourth Military Medical University. The experimental metastatic potential of HCC cells was assessed following intrasplenic injection as described previously [27].

### RNA interference

Cells were transfected with siRNAs using Lipofectamine 2000 (Invitrogen, Carlsbad, CA, USA) according to the manufacturer’s instructions. siRNAs targeting CD147 were designed and synthesized by Shanghai GenePharma Co. (Shanghai, China). The siRNA sequence is depicted in Table S1. GSK-3β siRNA was obtained from Santa Cruz Biotechnology (sc-35,527).

### Quantitative real-time PCR analysis

Total RNA was extracted using TRIzol reagents. Reverse transcription was performed using a PrimeScript RT reagent kit (TaKaRa Biotechnology, Japan). All primers were synthesized by BGI (BGI, Shenzhen, China) and their sequences are listed in Table S1. Quantitative real-time PCR (qRT-PCR) was performed using a SYBR Premix Ex Taq II Kit (TaKaRa Biotechnology, Japan).

### Luciferase reporter and TOPFlash/FOPFlash reporter assays

CTSB promoter (− 1100 ~ + 200) was cloned to pGL3-basic (pGL3-CTSB). pGL3-CTSB and pRL-TK (50:1) were cotransfected using Lipofectamine 2000. The luminescence was measured using Dual-luciferase reporter assay system and GloMax luminometer (Promega, WI, USA) according to the manufacturer’s instructions at 48 h after transfection. For the TOPFlash/FOPFlash reporter assay, TOPFlash and FOPFlash (Addgene, MA, USA) were cotransfected into cells along with siRNAs targeting CD147 or control siRNA. The data are represented as normalized TOPFlash/FOPFlash values. Experiments were performed in triplicate.

### Statistical analysis

Differences were compared by Student’s t test (two groups) or ANOVA followed by Tukey’s multiple comparisons test (three groups) as indicated in figure legends. Spearman R was used to determine correlations between relative expression of genes. Quantitative results are presented as mean values with SD. *P* < 0.05 was considered significant (GraphPad Prism 6, San Diego, CA).

## Results

### Elevated expression of CD147 correlates with collective invasion and predicts poor prognosis in patients with HCC

**Fig. 1:**
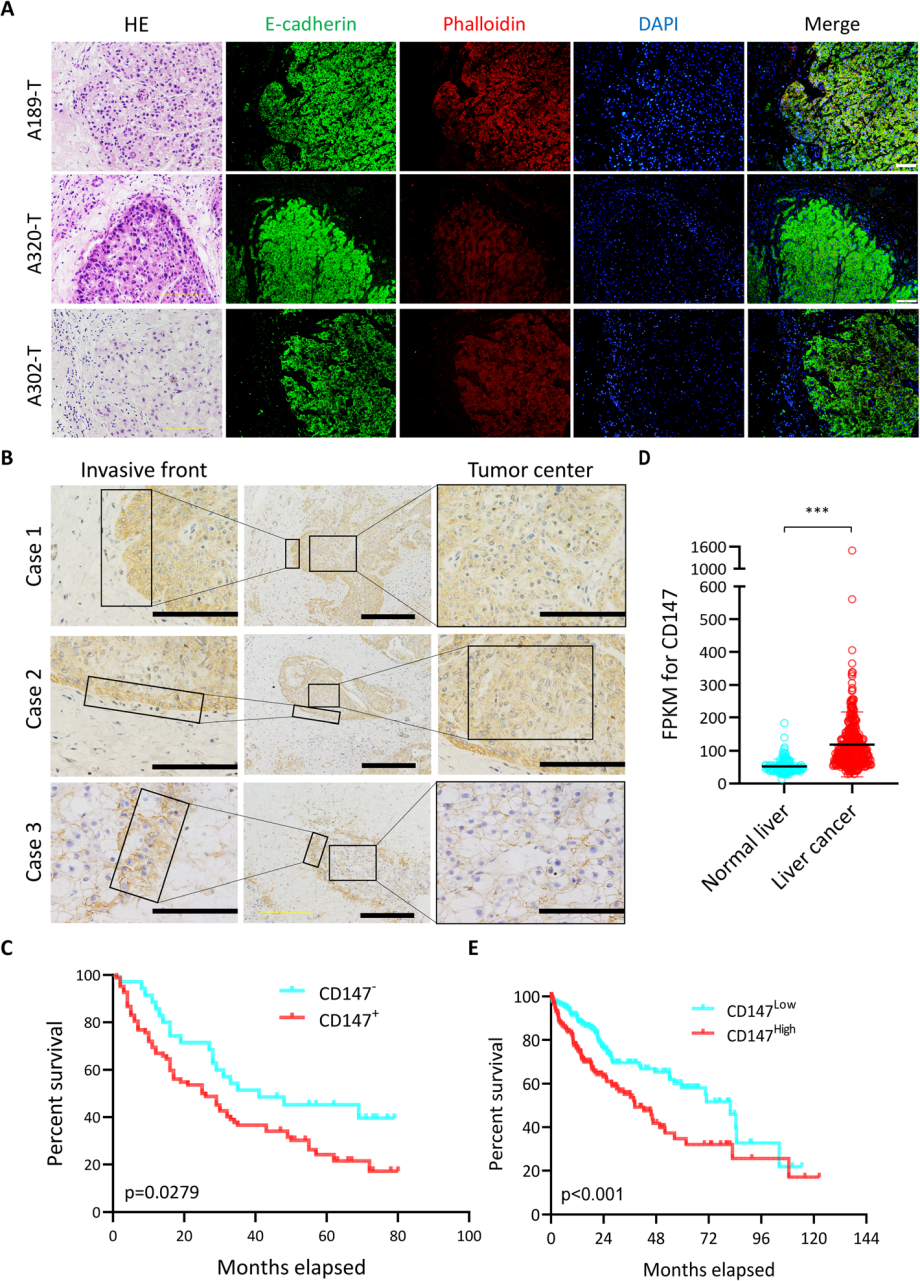
CD147 correlates with collective invasion in HCC. **a**. Representative images of HE staining (left) and immunofluorescent staining of E-cadherin, F-actin and nucleus of the invasive front of HCC tissues. Scale bar, 50 μm. **b**. Representative images of immunohistochemical staining of CD147 in invasive HCC tissues. Scale bar, 500 μm (middle), 200 μm (sides). **c**. Kaplan-Meier survival curves of HCC patients stratified into low- and high-expression of CD147 according to immunohistochemical staining. *P* value is determined by Log-rank (Mantel-Cox) test. **d**. CD147 expression in normal liver and liver cancer tissues. This data is derived from The Human Protein Atlas. *** *p* < 0.0001, Student’s t-test. **e**. Kaplan-Meier survival curves of HCC patients stratified into low- and high-expression of CD147. This data is derived from The Human Protein Atlas. P value is determined by Log-rank (Mantel-Cox) test

To investigate whether HCC cells predominantly undergo collective invasion in vivo, we analyzed the tumor specimens from HCC patients. We found that tumor cell groups were interconnected within the tumor mass. At the host-tumor interface, smaller groups of cells have penetrated the surrounding tissue (Fig. 1a). The presence of adherens junctions in conjunction with multicellular movement is a strong marker of collective invasion [14]. Immunofluorescence staining of E-cadherin revealed that the invading cells preserved E-cadherin expression and some leading cells have actin-rich protrusions extending into the ECM (Fig. 1a). These data indicated that HCC cells invade as multicellular units without a complete EMT signature.

As numerous studies have shown that CD147 regulates tumor invasion and metastasis, we checked the expression pattern of CD147 in HCC tissue specimens. We found that CD147 was predominantly expressed in the membrane (Fig. S1A). As previously reported, the expression of CD147 was increased in tumor cells compared with peritumoral liver tissue (Fig. S1B). Notably, the expression of CD147 was higher at the invasive front of tumor cell groups compared to the tumor center (Fig. 1b). Survival analysis showed that high expression of CD147 was significantly correlated with poor prognosis (Fig. 1c). Biopsies from an additional independent cohort immunostained by The Human Protein Atlas [28] confirmed that 1) CD147 is broadly upregulated at the protein level; 2) CD147 is prognostic, high expression is unfavourable in liver cancer (Fig. 1d-e). All these results suggest that CD147 is an unfavorable prognosis marker and elevated CD147 at the front of multicellular group undergoing collective invasion may be involved in collective invasion.

### CD147 promotes 3D collective invasion independent of MMPs induction

**Fig. 2:**
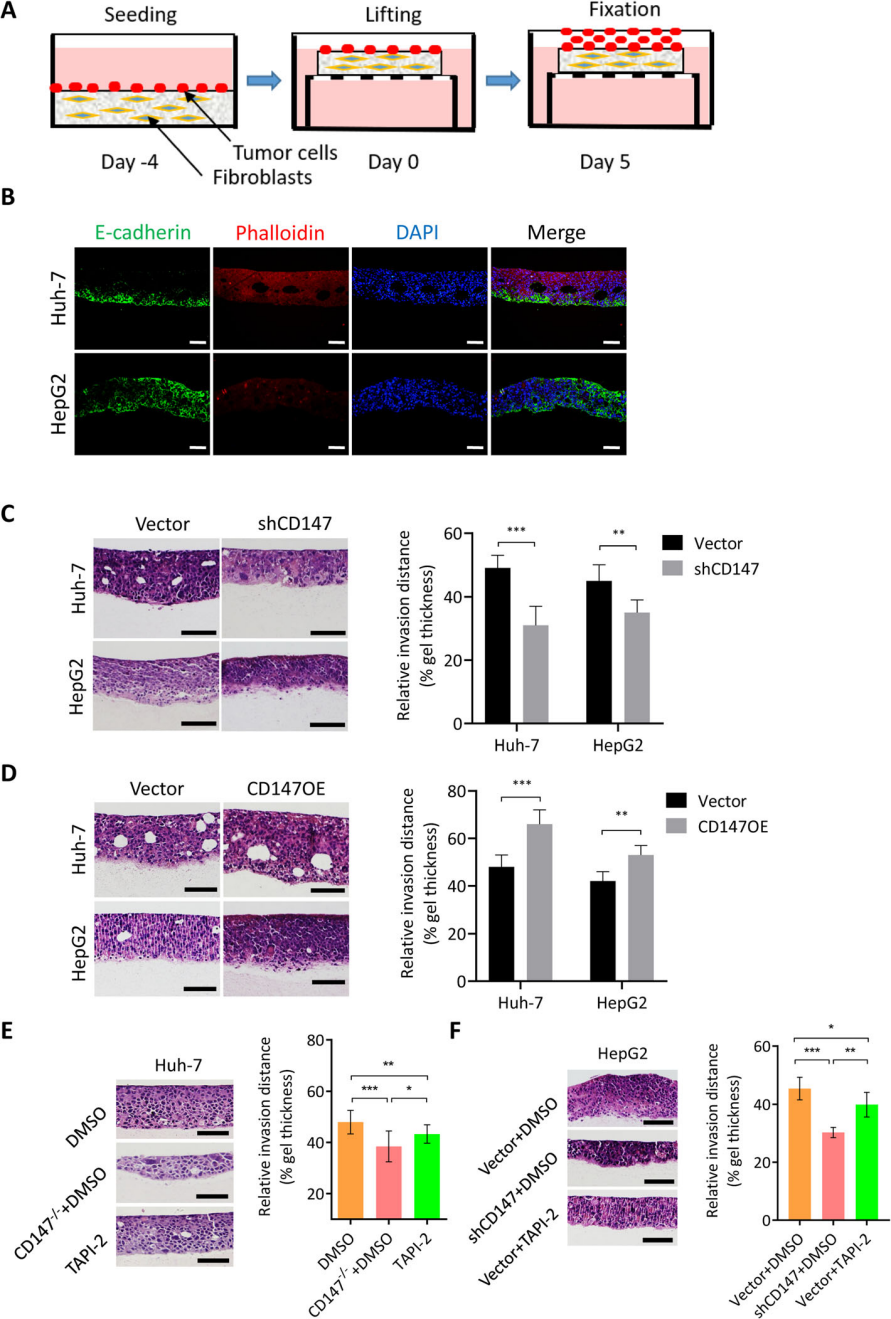
CD147-facilitated collective invasion is independent of MMP induction. **a**. Schematic diagram of in vitro 3D invasion model. **b**. Representative images of immunofluorescent staining of E-cadherin, F-actin and nucleus of 3D cultures from A. Scale bar, 50 μm. **c**-**d**. Representative images of HE staining of 3D cultures of Huh-7 and HepG2 cells transfected indicated constructs. Scale bar, 200 μm. Graph shows quantitative analysis of relative invasion distance. ** *p* < 0.01, *** *p* < 0.0001, Student’s t-test. **e**. Representative images of HE staining of 3D cultures of Huh-7 and CD147 knockout cells treated with or without TAPI-2. Scale bar, 200 μm. Graph shows quantitative analysis of relative invasion distance. * *p* < 0.05, ** p < 0.01, *** p < 0.0001, one-way ANOVA. **f**. Representative images of HE staining of 3D cultures of HepG2 and CD147 knockdown cells treated with or without TAPI-2. Scale bar, 200 μm. Graph shows quantitative analysis of relative invasion distance. * *p* < 0.05, ** p < 0.01, *** p < 0.0001, one-way ANOVA

To mimic the in vivo collective tumor cell invasion, we established a 3D invasion model (Fig. 2a). The HCC cells invaded through the collagen matrix as a cohesive, multicellular group and immunofluorescence staining of E-cadherin revealed that the invading cells preserved E-cadherin expression (Fig. 2b), which recapitulated the collective invasion of HCC in vitro. We then investigated the role of CD147 in collective invasion using this 3D invasion model. We found that silencing of CD147 attenuated cell invasion (Fig. 2c) and overexpression of CD147, by contrast, significantly boosted cell invasion (Fig. 2d) in both Huh-7 and HepG2 cells, which was consistent with the results from 2D invasion models [29]. CD147, also named extracellular matrix metalloproteinase inducer (EMMPRIN), is well known as a potent inducer of MMPs. To determine whether CD147 enhanced collective invasion was dependent entirely on MMPs induction, we treated cells with a broad-spectrum MMPs inhibitor TAPI-2 and found that CD147 still could significantly promote cell invasion though to a lesser extent (Fig. 2e-f). All these results suggest that MMPs are important but not unique downstream effectors for CD147 induced collective cell invasion.

### CD147 enhanced cathepsin B expression and activity

**Fig. 3:**
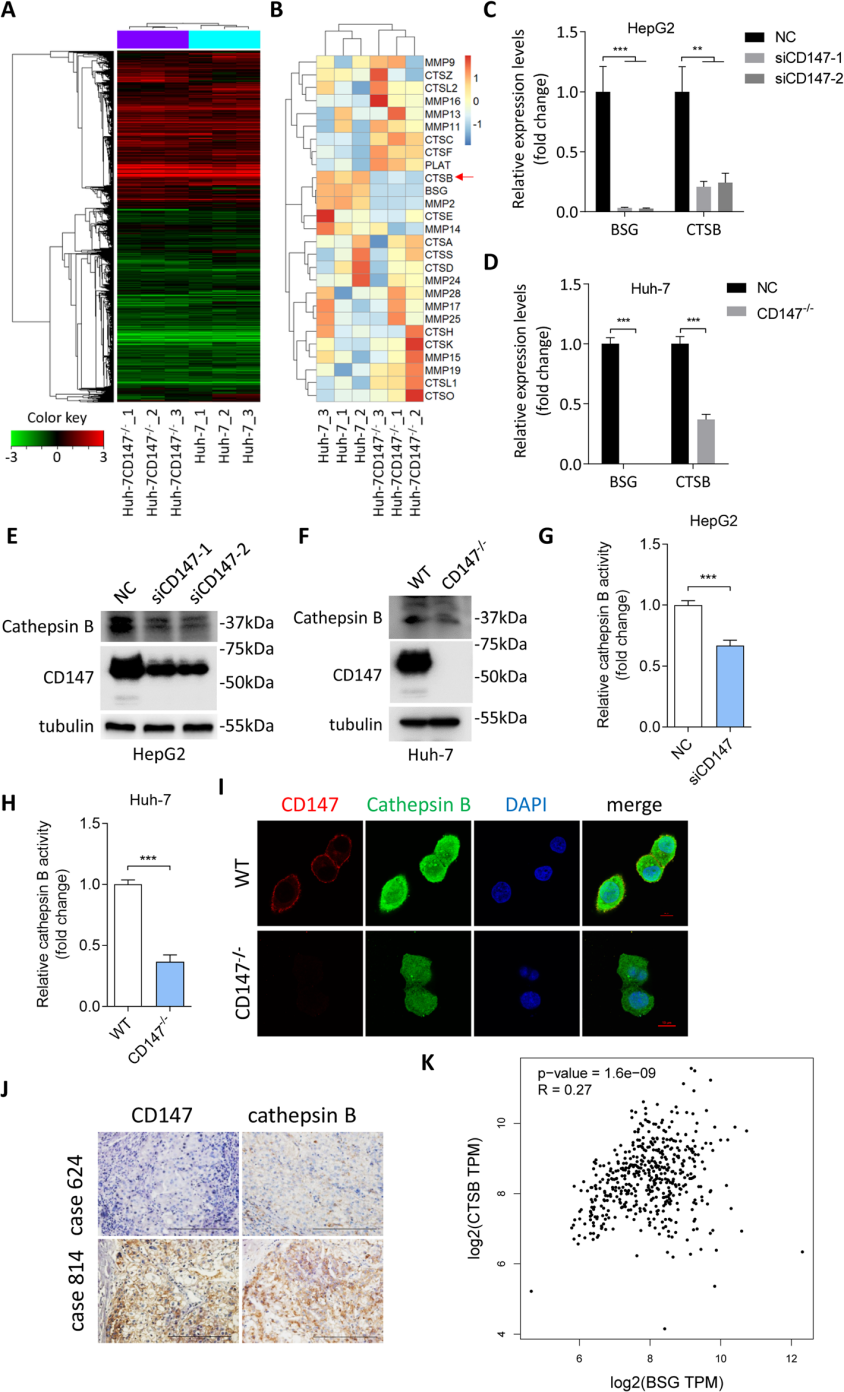
CD147 promotes cathepsin B expression and activation. **a**. Heatmap of differentially expressed genes between Huh-7 and CD147 knockout cells derived from 3D culture model. **b**. Expression profile of proteases in Huh-7 and CD147 knockout cells derived from 3D culture model. **c**. Quantitative real-time PCR analysis of BSG and CTSB expression in HepG2 cells transfected with siCD147 or negative control siRNA (NC). ** p < 0.01, *** p < 0.0001, one-way ANOVA. **d**. Quantitative real-time PCR analysis of BSG and CTSB expression in Huh-7 and CD147 knockout cells. *** p < 0.0001, Student’s t-test. **e**. Western blot analysis of indicated proteins in HepG2 cells transfected with siCD147 or negative control siRNA (NC). **f**. Western blot analysis of indicated proteins in Huh-7 and CD147 knockout cells. **g**. Quantitative analysis of cathepsin B activity in HepG2 cells transfected with siCD147 or negative control siRNA (NC). *** p < 0.0001, Student’s t-test. **h**. Quantitative analysis of cathepsin B activity in Huh-7 and CD147 knockout cells. *** p < 0.0001, Student’s t-test. **i**. Representative images of immunofluorescent staining of CD147, cathepsin B and nucleus. Scale bar, 10 μm. **j**. Representative images of immunohistochemical staining of CD147 and cathepsin B. Scale bar, 200 μm. **k**. Correlation analysis between BSG and CTSB using TCGA and GTEx data

Thus, we hypothesized that CD147 promotes ECM remodeling and collective cell invasion by regulating other factors, such as proteases besides MMPs. To examine this possibility, we performed transcriptome sequencing using total RNA extracted from cells in the 3D invasion model (Fig. 3a) and paid our attention to the expression profile of proteases (Fig. 3b). We found that MMP2 was decreased in CD147 knockout cells, which was consistent with the previous reports in various types of cancer [30, 31]. Besides MMP2, CTSB, which encodes cathepsin B protein, was significantly downregulated in CD147 knockout cells (*p* = 0.0002). We validated these results using qRT-PCR in HepG2 and Huh-7 cells. Silencing CD147 using siRNAs targeting CD147 resulted in significant reduction of CTSB mRNA expression in HepG2 cells (Fig. 3c) and CD147 knockout led to decreased CTSB mRNA expression in Huh-7 cells (Fig. 3d). We also checked cathepsin B protein expression and found that CD147 positively regulated cathepsin B protein expression in both HepG2 and Huh-7 cells (Fig. 3e-f). As cathepsin B functions mainly via its enzyme activity, we observed the effects of CD147 on cathepsin B activity utilizing the preferred cathepsin B substrate sequence RR labeled with amino-4-trifluoromethyl coumarin (AFC). We found that CD147 silence as well as CD147 knockout significantly downregulated cathepsin B activity (Fig. 3g-h). Subcellular localization is important for protein function, we found that CD147 knockout exerted little influence on subcellular location of cathepsin B, though the expression level of cathepsin B was significantly decreased (Fig. 3i). To ascertain whether the regulatory relationship between CD147 and cathepsin B did exist in HCC tissues, we analyzed CD147 and cathepsin B expression in HCC tissues by IHC and found that the expression of cathepsin B was significantly correlated with the expression of CD147 (Spearman r = 0.395, *p* = 0.0005, Fig. 3j and S2, Table 1). Bioinformatics analysis using TCGA and GTEx data confirmed that there was a positive correlation between CD147 and CTSB mRNA expression (Fig. 3k). All these results indicate that CD147 promotes cathepsin B expression and activation in HCC cells.

**Table 1:** Correlation between CD147 and cathepsin B expression in HCC tissues

CD147 expression	cathepsin B expression				Total, *N*
	0	1	2	3	
0	11	13	6	0	30
1	0	9	10	0	19
2	0	7	8	1	16
3	1	3	4	0	8
Total, *N*	12	32	28	1	73

Using the Spearman correlation analysis, ρ = 0.3954, p = 0.0005

### Cathepsin B is upregulated in HCC and promotes migration and invasion

**Fig. 4:**
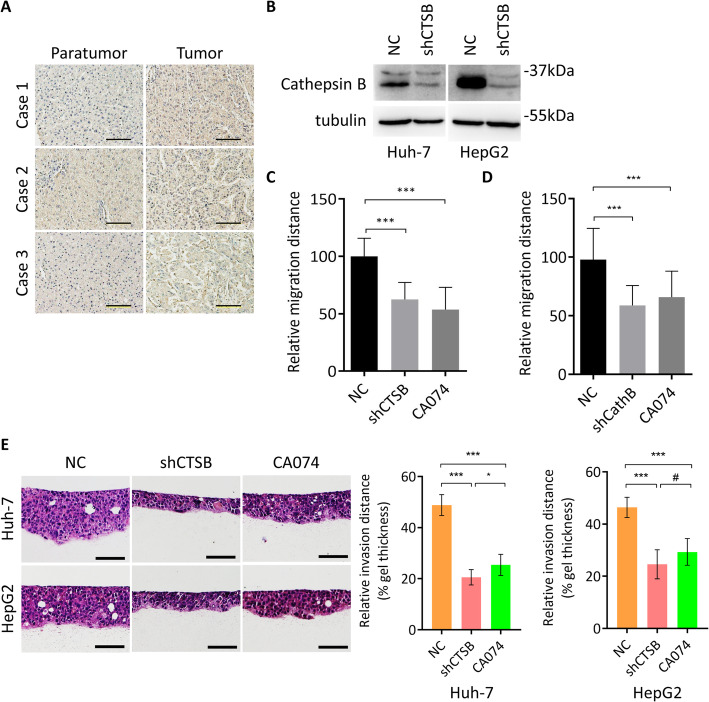
Enhanced cathepsin B promotes migration and collective invasion. **a**. Representative images of immunohistochemical staining of cathepsin B in paired tumor and paratumor tissues. Scale bar, 200 μm. **b**. Western blot analysis of cathepsin B in Huh-7 and HepG2 cells transfected with indicated constructs. **c**-**d**. Quantitative analysis of migration ability of Huh-7 (**c**) and HepG2 (**d**) cells transfected with indicated constructs or treated with CA074. *** *p* < 0.0001, one-way ANOVA. **e**. Representative images of HE staining of 3D cultures of Huh-7 and HepG2 cells transfected with indicated constructs or treated with CA074. Scale bar, 200 μm. Graph shows quantitative analysis of relative invasion distance. * *p* < 0.05, *** p < 0.0001, # *p* > 0.05, one-way ANOVA

To explore the potential role of cathepsin B in HCC, we first analyzed the expression pattern of cathepsin B in HCC tissues and paired paracancer tissues. We found that cathepsin B expression was increased compared with paracancer tissues (*p* = 0.0383, Fig. 4a), indicating that cathepsin B may be involved in HCC development. Next, we established cathepsin B stable knockdown cell lines (Fig. 4b) and evaluated the effects of cathepsin B knockdown and cathepsin B inhibitor CA074 on cell migration and 3D collective invasion. As shown in Fig. 4c-d, we found that cathepsin B knockdown as well as CA074 treatment significantly attenuated cell migration ability in HepG2 and Huh-7 cells. As expected, cathepsin B knockdown and CA074 treatment suppressed 3D collective invasion (Fig. 4e). All these results suggest that upregulated cathepsin B promotes cell migration and collective invasion in HCC.

### CD147-induced collective invasion depends on cathepsin B expression

**Fig. 5:**
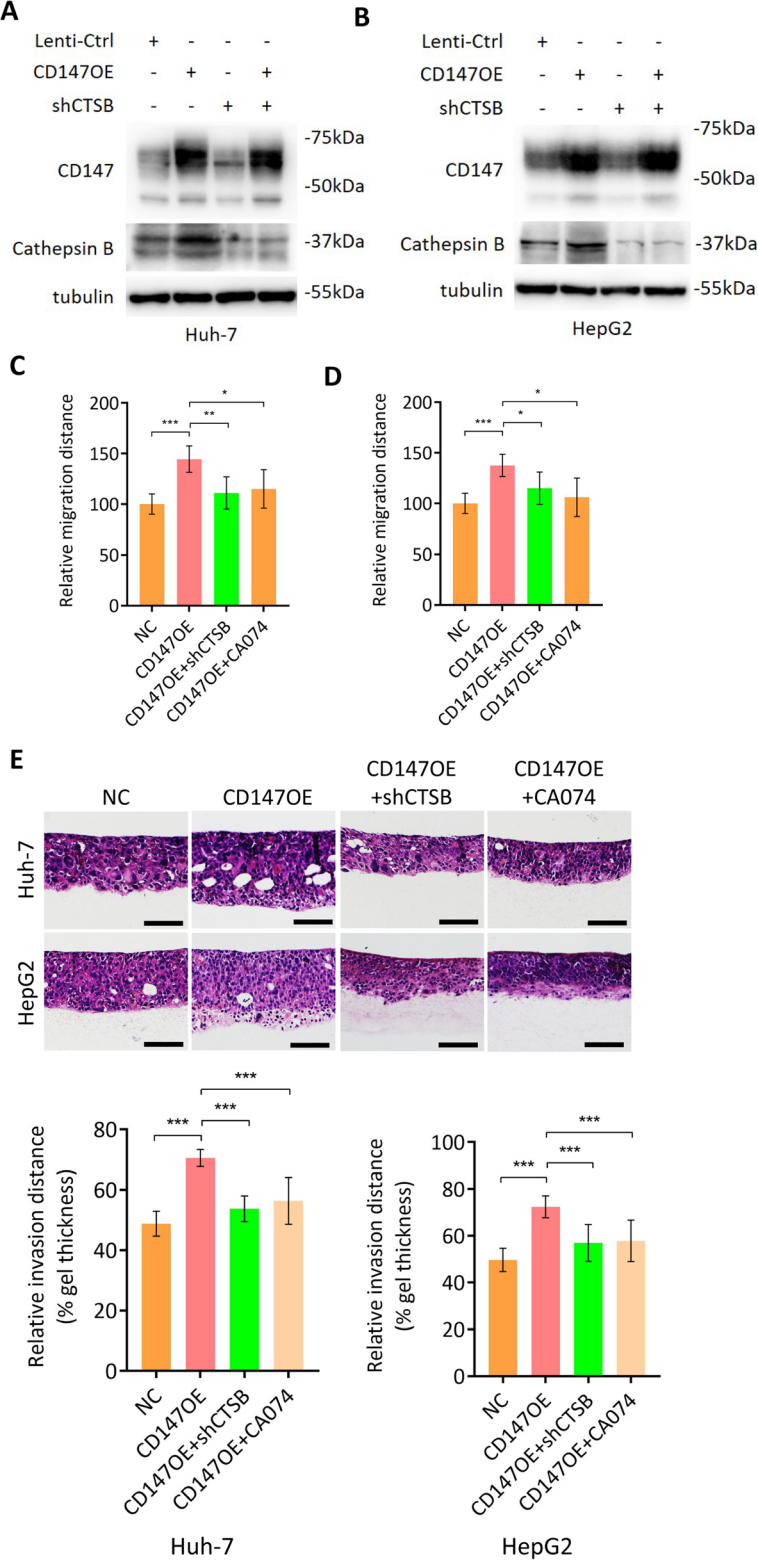
Cathepsin B mediates CD147-induced collective invasion. **a**-**b**. Western blot analysis of indicated proteins in Huh-7 (**a**) and HepG2 (**b**) cells transfected with indicated constructs. **c**-**d**. Quantitative analysis of migration ability of Huh-7 (**c**) and HepG2 (**d**) cells transfected with indicated constructs or treated with CA074. * p < 0.05, ** *p* < 0.01, *** p < 0.0001, one-way ANOVA. **e**. Representative images of HE staining of 3D cultures of Huh-7 and HepG2 cells transfected with indicated constructs or treated with CA074. Scale bar, 200 μm. Graph shows quantitative analysis of relative invasion distance. *** p < 0.0001, one-way ANOVA

Given CD147 enhances cathepsin B expression and cathepsin B promotes cell migration and collective invasion, it is not surprising to speculate that CD147 may facilitate cell migration and collective invasion via inducing cathepsin B expression. To examine this hypothesis, we first established HCC cell lines overexpressing CD147 while silencing CTSB (Fig. 5a-b). We found that overexpression of CD147 enhanced cell migration and the enhancement was significantly compromised by cathepsin B silence and CA074 treatment (Fig. 5c-d). As expected, knockdown of cathepsin B and CA074 treatment also impaired the promotive effects of forced expression of CD147 on collective invasion (Fig. 5e). All these results indicate that CD147 promotes cell migration and collective invasion at least partially by inducing cathepsin B expression.

### β-Catenin signaling regulates cathepsin B expression in HCC cells

**Fig. 6:**
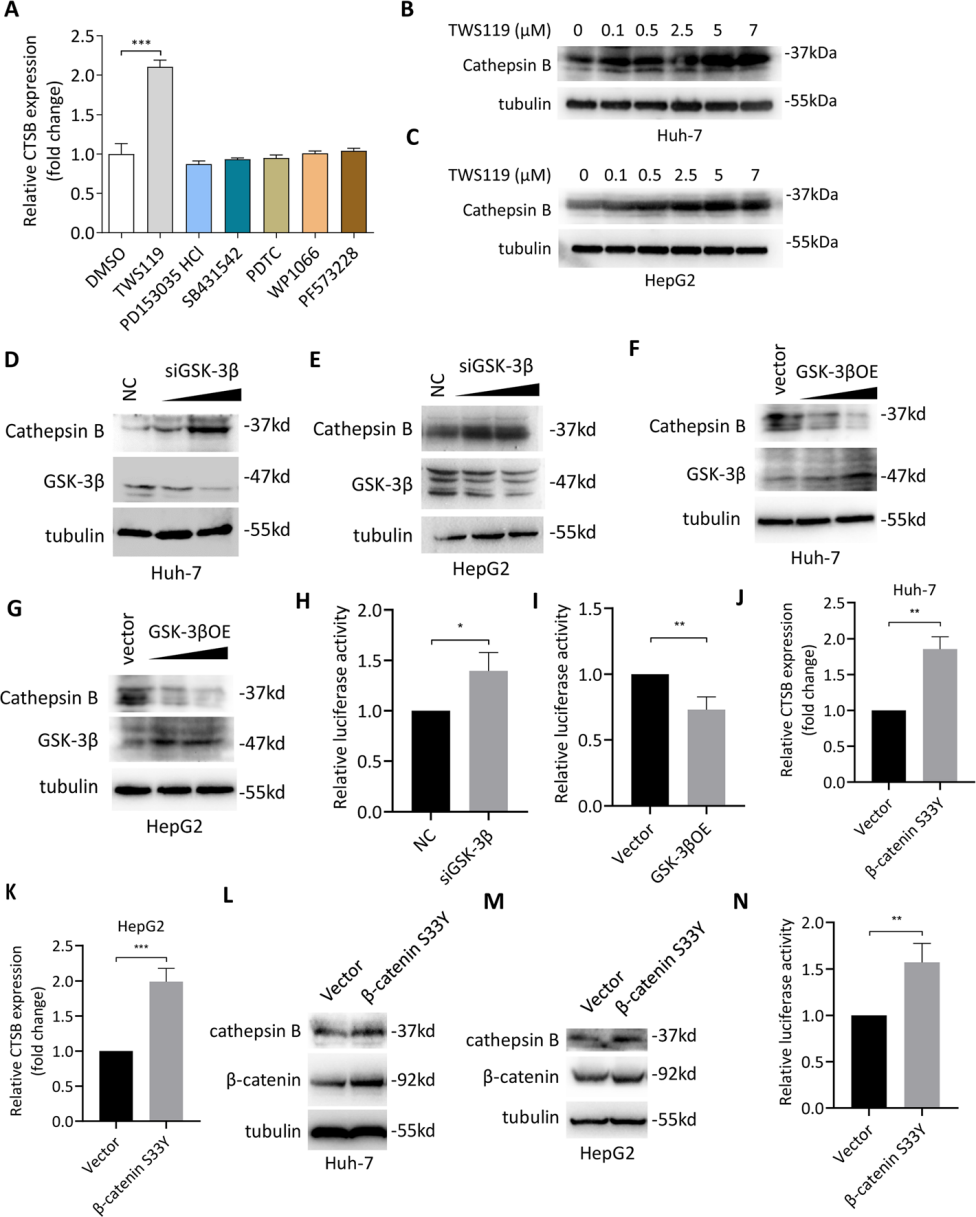
β-catenin signaling regulates cathepsin B expression. **a**. Quantitative real-time PCR analysis of CTSB expression in Huh-7 cells treated with indicated inhibitors. *** p < 0.0001, one-way ANOVA. **b**-**c**. Western blot analysis of cathepsin B in Huh-7 (**b**) and HepG2 (**c**) cells treated with increasing amounts of TWS119. **d**-**e**. Western blot analysis of indicated proteins in Huh-7 (**d**) and HepG2 (**e**) cells transfected with negative control siRNA (NC) or increasing amounts of siGSK-3β. **f**-**g**. Western blot analysis of indicated proteins in Huh-7 (**f**) and HepG2 (**g**) cells transfected with empty vector or increasing amounts of GSK-3β. **h**-**i**. Quantitative analysis of CTSB promoter luciferase reporter activity in Huh-7 cells transfected with siGSK-3β (**h**) or overexpressing GSK-3β (**i**). * p < 0.05, ** p < 0.01, Student’s t-test. **j**-**k**. Quantitative real-time PCR analysis of CTSB expression in Huh-7 (**j**) and HepG2 (K) cells transfected with empty vector or β-catenin S33Y. ** p < 0.01, *** p < 0.0001, Student’s t-test. **l**-**m**. Western blot analysis of indicated proteins in Huh-7 (**l**) and HepG2 (**m**) cells transfected with empty vector or β-catenin S33Y. **n**. Quantitative analysis of CTSB promoter luciferase reporter activity in Huh-7 cells transfected with empty vector or β-catenin S33Y. * p < 0.05, Student’s t-test

We next turned our attention to the mechanism by which CD147 regulates cathepsin B expression. To answer this question, we first screened the CD147-regulated signalings involved in cancer cell invasion via a panel of inhibitors. We found that TWS119, a specific GSK-3β inhibitor, could increase CTSB mRNA expression (Fig. 6a). Further analysis showed that TWS119 could increase cathepsin B protein expression in a dose-dependent manner (Fig. 6b-c). siRNA targeting GSK-3β resulted in enhanced cathepsin B expression (Fig. 6d-e) and overexpression of GSK-3β showed the opposite effects (Fig. 6f-g). To verify whether GSK-3β inhibitor TWS119 promotes cathepsin B expression at transcription level, we constructed a CTSB promoter-driven luciferase reporter and found that GSK-3β knockdown increased luciferase activity (Fig. 6h), while forced expression of GSK-3β generated the opposite effects (Fig. 6i). All these results indicate that GSK-3β regulates CTSB transcription in HCC.

As GSK-3β is recognized as a negative regulator of β-catenin signaling, we wondered whether CTSB is regulated by β-catenin signaling. We found that overexpression of β-catenin S33Y, a dominant positive mutant, led to increased CTSB mRNA expression in both Huh-7 and HepG2 cells (Fig. 6j-k). As expected, overexpression of β-catenin S33Y also increased cathepsin B protein expression (Fig. 6l-m) and CTSB promoter-driven luciferase activity (Fig. 6n). All these results indicate that β-catenin signaling promotes cathepsin B expression by enhancing CTSB transcription in HCC.

### CD147 promotes β-catenin signaling via reducing GSK-3β

**Fig. 7:**
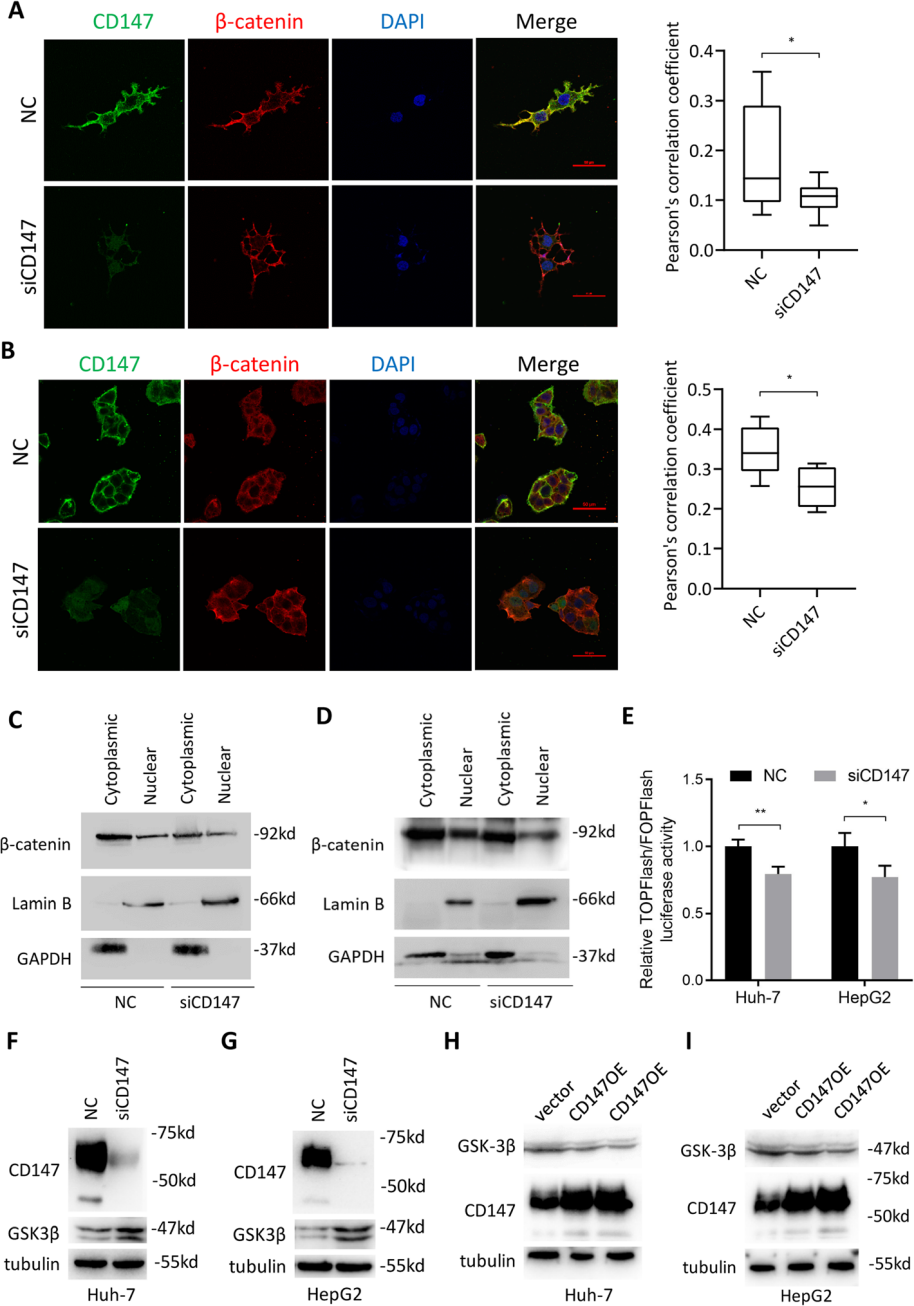
CD147 activates β-catenin signaling via downregulating GSK-3β. **a**-**b**. Representative images of immunofluorescent staining of CD147, β-catenin and nucleus (DAPI) in Huh-7 (**a**) and HepG2 (**b**) cells transfected with negative control siRNA or siCD147. Scale bar, 50 μm. Graph shows quantitative analysis of Pearson’s correlation coefficient between β-catenin and DAPI. * p < 0.05, Student’s t-test. **c**-**d**. Western blot analysis of indicated proteins in separated nuclear and cytosolic fragments in Huh-7 (**c**) and HepG2 (**d**) cells transfected with negative control siRNA or siCD147. **e**. The effect on TOP/FOP reporter activity in indicated cells transfected with negative control siRNA or siCD147 was proved by dual-luciferase assay. A Renilla transfection control normalized all results. ** p < 0.01, * p < 0.05, Student’s t-test. **f**-**g**. Western blot analysis of indicated proteins in Huh-7 (**f**) and HepG2 (**g**) cells transfected with negative control siRNA or siCD147. **h**-**i**. Western blot analysis of indicated proteins in Huh-7 (**h**) and HepG2 (**i**) cells transfected with empty vector or CD147

As we have shown that CTSB transcription is regulated by β-catenin signaling, we wondered whether CD147 regulates β-catenin signaling in HCC. β-catenin is recognized to tanslocalize to nucleus to activate target genes. We determined the effects of CD147 knockdown on β-catenin subcellular localization. We found that silencing CD147 led to decreased nuclear localization of β-catenin (Fig. 7a-b). To confirm these results, we compared the distribution of β-catenin between the nucleus and the cytoplasm. In consistent with the previous results, we found that the nuclear distribution of β-catenin was significantly reduced when CD147 was silenced (Fig. 7c-d). The transcriptional activity of TOP/FOP was significantly weakened in Huh-7 and HepG2 cells with downregulation of CD147 (Fig. 7e), indicating that CD147 knockdown resulted in decreased β-catenin signaling. Mechanically, we found that CD147 knockdown enhanced the expression of GSK-3β, a component of β-catenin destruction complex (Fig. 7f-g), and CD147 overexpression resulted in the opposite effects (Fig. 7h-i). All these results suggest that CD147 activates β-catenin signaling via suppressing GSK-3β expression.

### CD147 promotes cathepsin B expression via β-catenin signaling

**Fig. 8:**
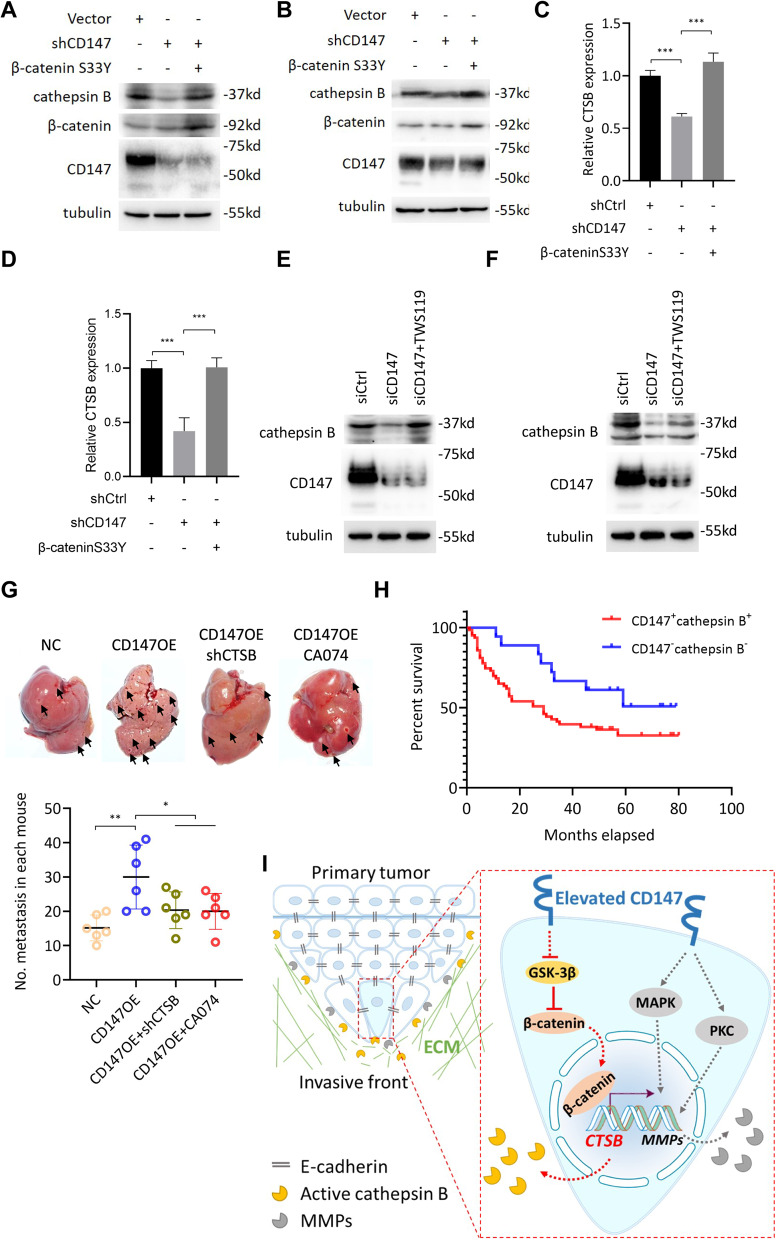
CD147 promotes cathepsin B via β-catenin signaling. **a**-**b**. Western blot analysis of indicated proteins in Huh-7 (**a**) and HepG2 (**b**) cells transfected with indicated constructs. **c**-**d**. Quantitative real-time PCR analysis of CTSB expression in Huh-7 (**c**) and HepG2 (**d**) cells transfected with indicated constructs. *** p < 0.0001, one-way ANOVA. **e**-**f**. Western blot analysis of indicated proteins in Huh-7 (b) and HepG2 (**f**) cells transfected with indicated siRNAs or treated with TWS119. G. Metastasis assay by intra-splenic injection of indicated cells in nude mice. Livers were excised for examination (left panel). Graph shows quantitative analysis of intrahepatic metastasis. * p < 0.05, ** p < 0.01, one-way ANOVA. **h**. Kaplan-Meier survival curves of HCC patients stratified into double positive- and double negative-expression of CD147 and cathepsin B according to immunohistochemical staining. **i**. Schematic representation of the major mechanisms of CD147 regulating collective invasion in hepatocellular carcinoma

As we have shown that β-catenin signaling regulates CTSB transcription (Fig. 6) and CD147 promotes β-catenin signaling (Fig. 7), we hypothesized that CD147 may upregulate cathepsin B expression via β-catenin signaling. To determine this possibility, we cotransfected HCC cells with β-catenin S33Y and siRNAs targeting CD147 and found that CD147 silence-induced cathepsin B protein (Fig. 8a-b) and CTSB mRNA (Fig. 8c-d) downregulation were restored by β-catenin S33Y overexpression. Similarly, GSK-3β inhibitor TWS119 also rescued CD147 silence-induced downregulation of cathepsin B expression (Fig. 8e-f). All these results suggest that β-catenin signaling is responsible for CD147-induced cathepsin B expression in HCC.

### CD147 enhances intrahepatic metastasis via cathepsin B

Enhanced cell migration and invasion are among key factors determining effective metastasis. As we have shown that CD147 promotes in vitro cell migration and collective invasion via inducing cathepsin B, we next established an in vivo intrahepatic metastasis model to evaluate the role of cathepsin B in CD147-induced in vivo metastasis. Consistent with the previous reports [24], we found that overexpression of CD147 increased intrahepatic metastasis, in sharp contrast, the enhancement of intrahepatic metastasis induced by CD147 overexpression was significantly compromised by CTSB silence as well as cathepsin B inhibition (Fig. 8g), indicating that cathepsin B is an important effector involved in CD147 enhanced HCC metastasis. The most common reason leads cancer patients to death is the metastasis, thus we analyzed the impacts of expression state of CD147 and cathepsin B on overall survival of patients with HCC. As shown in Fig. 8h, we found that the prognosis of patients with high expression of CD147 and cathepsin B was unfavourable in HCC. These results suggest that cathepsin B contributes to CD147-promoted HCC invasion and metastasis (Fig. 8i) and the coexpression of CD147 and cathepsin B is a poor prognosis factor.

## Discussion

Most recently, mounting evidence has shown that cancer cells can invade as a cohesive and multicellular group, which is called collective invasion [32]. Collective invasion is different from single cell invasion, which depends on cell-type-specific mechanisms and induction by the microenvironment [33] and contributes to carcinogenesis, progression and distant dissemination of various kinds of cancers including lung cancer, breast cancer, soft tissue sarcomas and melanoma [14, 34, 35]. Whether HCC cells adopt collective invasion in the process of metastasis is uncertain. In this study, we showed that HCC cells also can invade as a cohesive and multicellular group by analyzing tumor tissues from patients with HCC (Fig. 1). Numerous studies have shown that EMT is implicated in HCC progression and metastasis [36], we also did observe that some tumor cells in the front of the tumor mass showed a mesenchymal phenotype with expression deficiency of E-cadherin in HCC tissues (data not shown). Therefore, it is safe to conclude that HCC cells may adopt collective invasion as well as other forms of invasion in progression and metastasis.

During tumor progression, the stroma, which surrounds the epithelial tissues, can significantly impact the mode and direction of tumor cell invasion. ECM may both initiate and drive disease progression. The ECM is a complex grid consisting of multiple proteins, most of which play a vital role in containing the essential information needed for maintenance of a sophisticated structure anchoring the cells and sustaining normal function of tissues. The application of 3D models composing of human cells and ECM in cancer research has grown in the last two decades [37, 38] to provide in vitro models with biological relevance. Such studies have cast new light on basic cancer biology and re-emphasized how the tumor microenvironment dictates cancer fate. Collagen type I is the major ECM protein in liver and its increased expression has been associated with cancer progression [39]. In this study, to stablish a more realistic 3D invasion model, collagen type I was used as the major ECM protein and fibroblasts were employed as the stromal elements and the main source of chemokine (Fig. 2a). Using this 3D invasion model, we found that HCC cells invaded into the ECM as a cohesive and multicellular group with E-cadherin mediating cell junctions (Fig. 2b), which is consistent with the observation that tumor cells can invade collectively in HCC tissues.

Most importantly, we found that CD147 could facilitate collective invasion. Previous studies have shown that CD147 promotes tumor cell adhesion, migration and invasion in various kinds of cancers [40] and the underlying mechanisms are diverse depending on the type of cancer and are not fully understood. We and others have found that enriched expression of CD147 on the surface of tumor cells induces stromal and tumor cells themselves to increase MMPs expression and secretion, leading to the degradation of the surrounding stroma [30, 41]. Though, CD147 was shown to induce MMP2 [42], MMP9 [41], MMP11 [43], MMP13 and MMP14 [44] in liver and our previous work proved that 3D co-culture of HCC cells and fibroblasts using matrigel enhanced CD147 expression, which in turn caused increased production of MMP2 and MMP9 [45], unexpectedly, we found that the supportive effect of CD147 on collective invasion was not abolished by a broad-spectrum MMPs inhibitor (Fig. 2) in 3D invasion model. As invasion is a multistep and complex process: adhesion, migration and degradation of stroma all contribute to effective tumor cell invasion, it is not surprising to conclude that MMPs induction may play a minor role in CD147 facilitated 3D invasion.

Cathepsin B is a cysteine proteolytic enzyme which has a low expression level in normal tissues, and is predominantly located in lysosomes. Cathepsin B is strictly regulated at multiple levels under physiological conditions and expressed in the form of inactive zymogens. Its activation requires a slightly acidic environment such as lysosomes. However, in the pathological state, cathepsin B is released by the cells involved in a variety of pathological processes. The study found that the expression and activation of cathepsin B were both upregulated in breast cancer, lung cancer, melanoma, gastric cancer, colon cancer and other malignant tumors [46]. Here we found that cathepsin B was elevated in HCC tissues compared with adjacent normal liver tissues. Tumor cells release cathepsin B through microbubbles, exosomes, etc. Krueger S and colleagues have found that interfering with cathepsin B expression can reduce the movement and invasion of osteosarcoma cells [47]. A negative correlation of cathepsin B expression and laminin in gastric [48] and colorectal [49] cancer suggests the involvement of cathepsin B in ECM remodeling. It has also been found that downregulating of cathepsin B expression in mouse breast cancer models can inhibit type I collagen degradation and bone metastasis [50]. Joyce et al. used cathepsin B knockout mice to detect the function of pancreatic islet cell tumor, in which the proliferation, angiogenesis and invasion of cells were decreased, while cell apoptosis was increased [51]. Therefore, cathepsin B which releases to extracellular compartment has a multifaceted effect on tumor cells: activation of cathepsin B on the one hand can hydrolyze ECM and activate matrix metalloproteinases, triggering ECM remodeling, promoting tumor migration and invasion. On the other hand, cathepsin B can activate a variety of growth factors or increase its release, promoting angiogenesis and tumor proliferation [52].

The signaling cascades upstream of cathepsin B are diverse in various tumors. Complement C1q tumor necrosis factor-related protein 8 (CTRP8) interacts with RXFP1 and induces the production and secretion of cathepsin B in glioblastoma cells, leading to laminin degradation and glioblastoma dissemination [53]. Epidermal growth factor cytoplasmic domain (EGFcyt) was identified as an inducer of lysosomal cathepsin B expression in thyroid and glioma carcinoma cells [54]. Metastasis-associated protein (MTA1) inhibits cathepsin B expression in prostate cancer [55]. By mRNA sequencing and functional validation, CD147 was identified as a novel regulator of cathepsin B, which mediated CD147-faciliated collective invasion in HCC. Via analyzing serial slices, we found that the expression of cathepsin B was positively correlated with CD147. As to molecular mechanism, we found that CD147 downregulated GSK-3β and stabilized β-catenin, which enhanced CTSB transcription. Sidhu SS et al. found that CD147 enhanced β-catenin signaling in lung cancer [56]. Knutti N et al. showed that a feed-forward circuit existed between β-catenin signaling and CD147 expression in breast cancer [57]. Our previous data showed that overexpression of CD147 promoted β-catenin nuclear translocation in HCC [24, 58]. And CD147 was also reported to modulate androgen receptor activity through β-catenin pathway in prostate cancer [59]. Together with the data reported here, these results indicate that β-catenin signaling is key downstream pathway which mediates divers tumor promotion effects of CD147. Qiang Wu et al. observed that siRNA inhibition of β-catenin leaded to decreased CTSB mRNA expression in placental trophoblast [60]. In contrast, cathepsin B was reported to promote porcine preadipocytes differentiation by degrading fibronectin and attenuating the β-catenin signaling pathway [61]. This discrepancy indicates that the molecular regulation of cathepsin B is cell context specific.

In summary, our novel findings provided an important piece of evidence regarding the critical role of CD147 in HCC cells collective invasion and implied that targeting CD147 would be valuable for the development of novel therapeutic modalities against invasion and metastasis of cancer.

## Conclusions

Enhanced expression of CD147 at the invasive front of HCC cell groups promoted HCC cells collective invasion via upregulating cathepsin B expression and targeting CD147 would be valuable for the development of novel therapeutic modalities against invasion and metastasis of cancer.

## Supplementary information

**Additional file 1: Figure S1.** CD147 expression in paired tumor and paratumor tissues. A. Representative images of immunohistochemical staining of CD147 in paired tumor and paratumor tissues. Scale bar, 200 μm. B. Heatmap shows IHC score of CD147 expression in paired tumor and paratumor tissues.

**Additional file 2: Figure S2.** Serial sections of HCC tissues were stained with immunohistochemistry and heatmap shows IHC score of CD147 and cathepsin B.

**Additional file 3: Table S1**. Oligo sequences.

## Data Availability

The datasets and materials used during the current study are available from the corresponding author on reasonable request.

## Abbreviations

DEGs: Differentially expressed genes
DMEM: Dulbecco’s Modified Eagle’s medium
ECM: Extracellular matrix
EMT: Epithelial-to-mesenchymal transition
FBS: Fetal bovine serum
HCC: Hepatocellular carcinoma
HE: Hematoxylin and eosin
IHC: Immunohistochemical
MMP: Matrix metalloproteinase
