# Correction: CD147 promotes collective invasion through cathepsin B in hepatocellular carcinoma

**DOI:** 10.1186/s13046-024-03246-x

**Published:** 2024-12-16

**Authors:** Shi-Jie Wang, Dong Chao, Wei Wei, Gang Nan, Jia-Yue Li, Fen-Ling Liu, Ling Li, Jian-Li Jiang, Hong-Yong Cui, Zhi-Nan Chen

**Affiliations:** 1 National Translational Science Center for Molecular Medicine, Department of Cell Biology, Fourth Military Medical University, Xi’an, 710032 P. R. China; 2 Department of thoracic surgery, The 940th hospital of joint logistics support force of Chinese People’s Liberation Army, Lanzhou, 730050 P. R. China

**Correction: J Exp Clin Cancer Res 39, 145 (2020)**

10.1186/s13046-020-01647-2

Following publication of the original article [1], the author identified errors in Fig. 8. The western blot bands of Cathepsin B in Fig. 8F and the representative image for the CD147OE + shCTSB group in Fig. 8G were erroneously assembled.

The corrected figure is presented below:

**Incorrect Fig. 8**

**Fig. 8:**
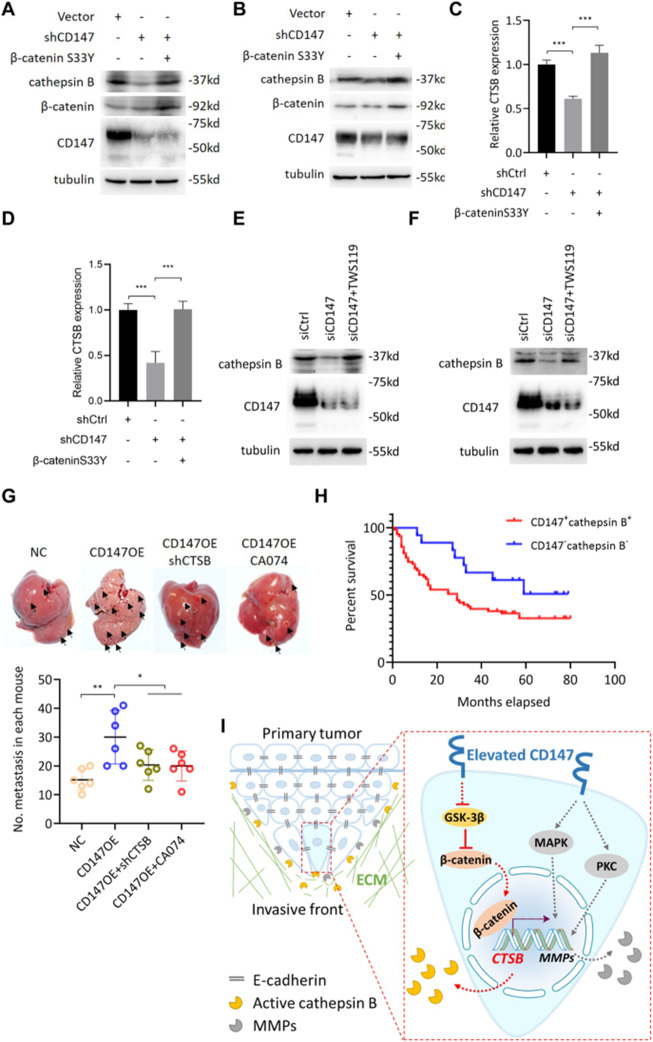
CD147 promotes cathepsin B via β-catenin signaling. **a-b**. Western blot analysis of indicated proteins in Huh-7 (**a**) and HepG2 (**b**) cells transfected with indicated constructs. **c-d**. Quantitative real-time PCR analysis of CTSB expression in Huh-7 (**c**) and HepG2 (**d**) cells transfected with indicated constructs. *** *p* < 0.0001, one-way ANOVA. **e-f**. Western blot analysis of indicated proteins in Huh-7 (b) and HepG2 (**f**) cells transfected with indicated siRNAs or treated with TWS119. G. Metastasis assay by intra-splenic injection of indicated cells in nude mice. Livers were excised for examination (left panel). Graph shows quantitative analysis of intrahepatic metastasis. * *p* < 0.05, ** *p* < 0.01, one-way ANOVA. **h**. Kaplan-Meier survival curves of HCC patients stratified into double positive- and double negative-expression of CD147 and cathepsin B according to immunohistochemical staining. **i**. Schematic representation of the major mechanisms of CD147 regulating collective invasion in hepatocellular carcinoma

**Correct Fig. 8**

**Fig. 8:**
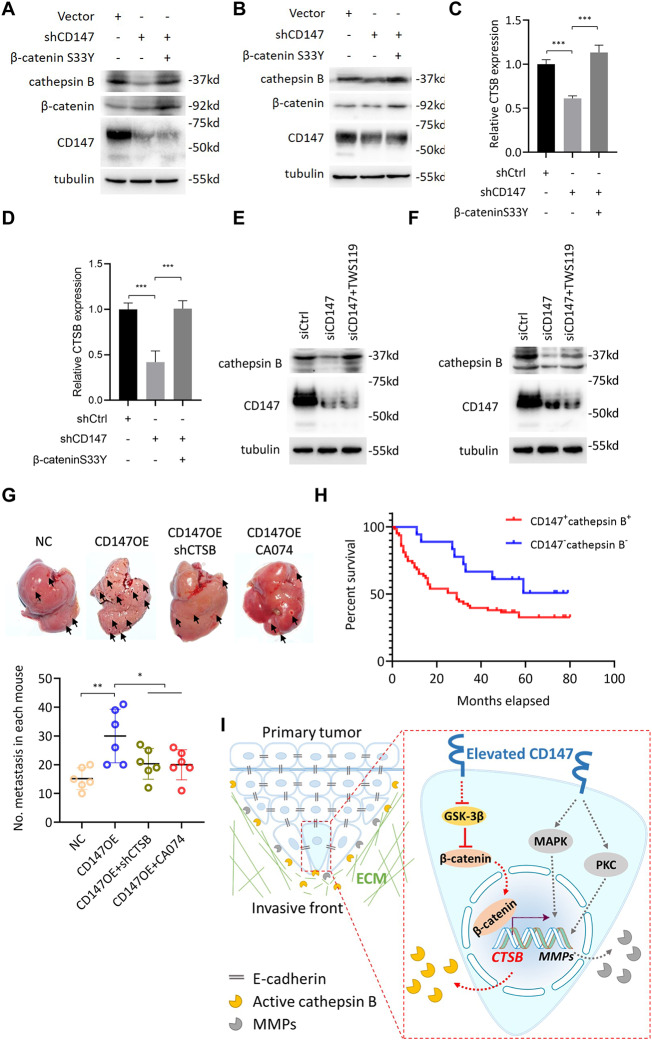
CD147 promotes cathepsin B via β-catenin signaling. **a-b**. Western blot analysis of indicated proteins in Huh-7 (**a**) and HepG2 (**b**) cells transfected with indicated constructs. **c-d**. Quantitative real-time PCR analysis of CTSB expression in Huh-7 (**c**) and HepG2 (**d**) cells transfected with indicated constructs. *** *p* < 0.0001, one-way ANOVA. **e-f**. Western blot analysis of indicated proteins in Huh-7 (b) and HepG2 (**f**) cells transfected with indicated siRNAs or treated with TWS119. G. Metastasis assay by intra-splenic injection of indicated cells in nude mice. Livers were excised for examination (left panel). Graph shows quantitative analysis of intrahepatic metastasis. * *p* < 0.05, ** *p* < 0.01, one-way ANOVA. **h**. Kaplan-Meier survival curves of HCC patients stratified into double positive- and double negative-expression of CD147 and cathepsin B according to immunohistochemical staining. **i**. Schematic representation of the major mechanisms of CD147 regulating collective invasion in hepatocellular carcinoma

The corrections do not affect the overall results, discussion, or conclusion of the article.
