# Supplementary figures and images for: CD147 promotes collective invasion through cathepsin B in hepatocellular carcinoma
Source: J Exp Clin Cancer Res. 2020 Jul 29;39:145. doi: 10.1186/s13046-020-01647-2 (PMC7391525; doi:10.1186/s13046-020-01647-2)

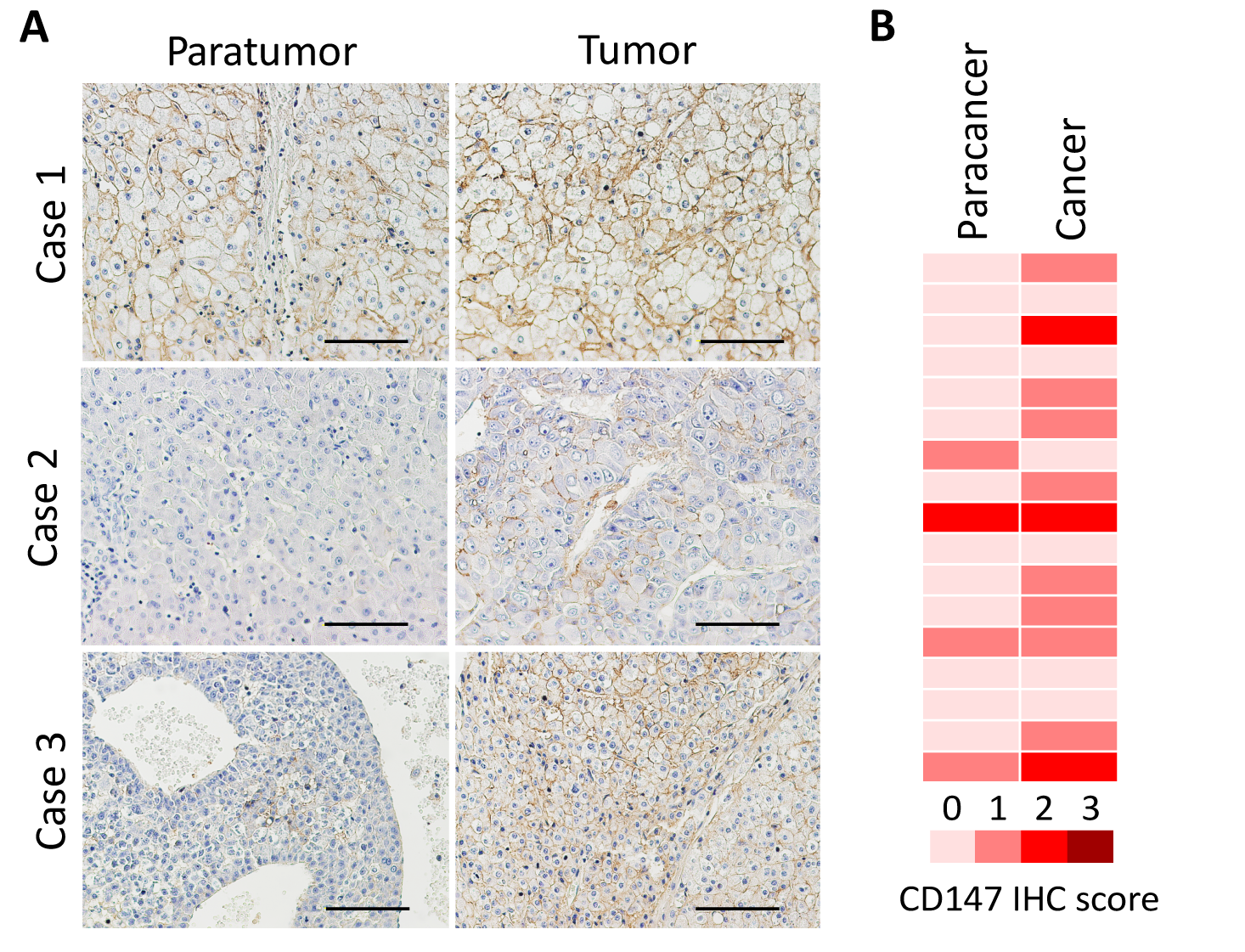

Supplement: Supplementary file 1 — Additional file 1: Figure S1. CD147 expression in paired tumor and paratumor tissues. A. Representative images of immunohistochemical staining of CD147 in paired tumor and paratumor tissues. Scale bar, 200 μm. B. Heatmap shows IHC score of CD147 expression in paired tumor and paratumor tissues. [file 13046_2020_1647_MOESM1_ESM.tif]

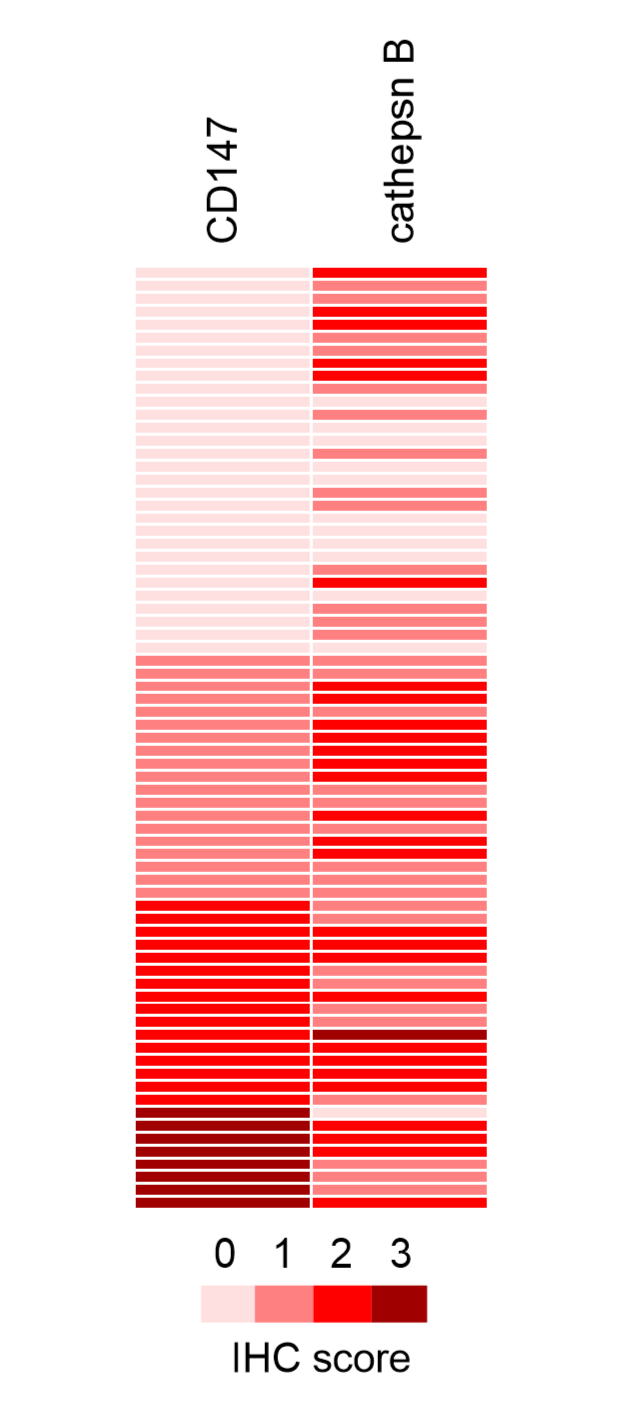

Supplement: Supplementary file 2 — Additional file 2: Figure S2. Serial sections of HCC tissues were stained with immunohistochemistry and heatmap shows IHC score of CD147 and cathepsin B. [file 13046_2020_1647_MOESM2_ESM.tif]
