# Supplementary material for: CD147 promotes collective invasion through cathepsin B in hepatocellular carcinoma
Source: J Exp Clin Cancer Res. 2020 Jul 29;39:145. doi: 10.1186/s13046-020-01647-2 (PMC7391525; doi:10.1186/s13046-020-01647-2)
Supplement: Supplementary file 3 — Additional file 3: Table S1. Oligo sequences. [file 13046_2020_1647_MOESM3_ESM.docx]

| Table S1 Oligo sequences | | |
| --- | --- | --- |
| Real-time RT-PCR primers | | |
| CD147 | Forward | 5’-ACTCCTCACCTGCTCCTTGA-3’ |
|  | Reverse | 5’-GCCTCCATGTTCAGGTTCTC-3’ |
| CTSB | Forward | 5’-ACAGGCCATGTGAGCCACCG-3’ |
|  | Reverse | 5’-CGCTTTCCATTCCTGCGTCTCTGTCTTG-3’ |
| GAPDH | Forward | 5’-GCACCGTCAAGGCTGAGAAC-3’ |
|  | Reverse | 5’-TGGTGAAGACGCCAGTGGA-3’ |
| siRNA oligos | | |
| siCD147-1 | Sense | 5′-CCACUUUGGUGUUUCAUAAtt-3′ |
| siCD147-2 | Sense | 5'-GUUCUUCGUGAGUUCCUCtt-3' |
| siCTSB | Sense | 5′-GCAUGAUUCUUUAAUAGAATT-3′ |
| Mutant primers |  |  |
| β-cateninS33Y | Forward | 5'-cagaatggattccatagtccaggtaagactgttgct-3 |
|  | Reverse | 5'-agcaacagtcttacctggactatggaatccattctg-3' |
